# Does SuperPark Make Children Less Sedentary? How Visiting a Commercial Indoor Activity Park Affects 7 to 12 Years Old Children’s Daily Sitting and Physical Activity Time

**DOI:** 10.3390/ijerph15081595

**Published:** 2018-07-27

**Authors:** Arto J. Pesola, Martti Melin, Anssi Vanhala, Ying Gao, Taija Finni

**Affiliations:** 1 Active Life Lab, South-Eastern Finland University of Applied Sciences, FI-50101 Mikkeli, Finland; 2 Faculty of Sport and Health Sciences, University of Jyväskylä, FI-40014 Jyväskylä, Finland; martti.m.melin@student.jyu.fi (M.M.); anssi.o.vanhala@student.jyu.fi (A.V.); ying.y.gao@jyu.fi (Y.G.); taija.m.juutinen@jyu.fi (T.F.)

**Keywords:** sitting, moderate-to-vigorous physical activity, indoor activity park, children, accelerometer

## Abstract

Commercial indoor activity parks provide children with a variety of entertaining physical activities. This study examined whether visiting SuperPark affects total daily sitting and physical activity time. The participants (8 girls and 7 boys, aged 10.3 ± 1.9 years, height 144.5 ± 11.8 cm, body mass index (BMI) 19.3 ± 3.0 kg/m^2^) wore a thigh-worn accelerometer during a normal week and were provided free tickets to visit SuperPark on at least one day. On average, the children spent 3.3 ± 1.2 h in SuperPark. During the visits the children had 0.9 h less sitting (0.7 ± 0.3 h, *p* = 0.000) and 0.9 h more moderate-to-vigorous physical activity (MVPA; 1.4 ± 0.6 h, *p* = 0.002) as compared to the reference periods on days without a SuperPark visit (1.6 ± 0.3 h sitting and 0.5 ± 0.4 h MVPA). During the days when visiting SuperPark, sitting time decreased 1.0 h (5.8 ± 0.9 h, *p* = 0.008) and MVPA increased 0.8 h (3.0 ± 1.0 h, *p* = 0.017) as compared to the reference days (6.8 ± 1.1 h sitting and 2.2 ± 0.8 h MVPA). The effects were more pronounced during weekdays than weekends. The children spent more than three hours in SuperPark on one visit, of which almost a half was MVPA. During the whole day, one hour of sitting was replaced with MVPA, suggesting that visiting SuperPark has the potential to improve health. Whether children continue visiting SuperPark and gain health benefits merits investigation.

## 1. Introduction

The physical activity milieu of children has changed over the past decades. The number of children engaging in organized physical activities has either increased or remained the same, whereas unorganized forms of physical activities, like active transport, have decreased [1,2,3]. Less than half of Finnish primary school children reach the recommended 60 min of moderate-to-vigorous physical activity (MVPA) daily and remain sedentary for 65% of their waking time [4,5]. Insufficient level of childhood physical activity is associated with metabolic risks and predicts development of metabolic diseases in adulthood [6,7,8]. Excess sedentary behavior, defined as any waking behavior in a sitting/reclining posture with low energy expenditure, exacerbates the risks directly and/or indirectly by replacing the physical activities [9,10].

In the modern environment, ubiquitous access to sedentary pursuits may compete with children’s’ physical activity, especially in girls and in obese children [11,12]. Making physical activity fun and letting children choose their preferred forms of physical activities are methods to increase motivation towards and participation in physical activities [13,14]. However, increasing physical activity while decreasing sedentary behavior during the whole day is not straightforward. At a given time-point, children can either be active or sedentary. The more fun and reinforcing the physical activity, the more likely and the longer children are willing to engage in it instead of choosing the sedentary alternative [15]. However, participating in physical activities at a given time does not necessarily reduce the sedentary time during the whole day [13,16]. The children may compensate for the increased activity time by being more sedentary outside of the physical activity session [17,18]. Because the health benefits children obtain are dependent on the total daily physical activity and sedentary behaviors, it is important to consider how these behaviors interact following participation in different forms of physical activities.

Having access to additional forms of physical activities would possibly prevent a decline in children’s total physical activity time. Several indoor activity park concepts have been introduced during the past years in Finland. For example, SuperPark Ltd. markets to provide a place for children and adults “to have fun, experience happiness and connect with friends” [19]. Since its establishment in 2012, SuperPark Ltd. has opened 13 parks in Finland and one in Hong Kong, China. In 2018, they expect to have 2 million customers and aim to open 100 parks by 2022 [19]. These numbers illustrate a demand for commercial physical activity supply, which is fun and enjoyable. Despite the potential of commercial activity parks to reduce sitting time and to increase physical activity time during the whole day, this has not been shown with objective monitoring, which was the aim of this study. By measuring habitual sitting and physical activity of children, this study tested the hypothesis that visiting SuperPark decreases sitting time and increases MVPA during the SuperPark visits and the whole day.

## 2. Participants and Methods

### 2.1. Study Design and Procedure

This study was carried out in the city of Jyväskylä, Finland, where the SuperPark Jyväskylä is located near the city center and can be accessed by foot, cycle, car, or public transport. A within subject design was utilized, where the participants’ habitual sitting and physical activity were compared between the days when they visited SuperPark to days without a SuperPark visit. A convenience sample of 15 children was recruited by randomly asking SuperPark Jyväskylä visitors, which were in the SuperPark during recruitment, to participate voluntarily. Inclusion criteria were 7–12-year-old children without any limitations for participating in vigorous physical activities and without regular (more than once per week) systematic vigorous sporting activity (like playing in a football team). Every interested and eligible participant or their parents were briefed about the protocol and gave an informed consent. The children that were in the SuperPark without their parents were required to contact their parents for permission. The ethics committee of University of Jyväskylä approved the study (JYU 10.08.2017) and participants’ parents signed an informed consent prior to the measurements. Data were collected in August–September 2017.

The participants’ parents were asked to schedule the first laboratory measurement on a day that initiated a seven-day measurement period, which would represent the child’s typical everyday life (e.g., the child goes to school normally). The laboratory measurements assessed body composition after overnight fast and initiated the seven-day activity measurement period. During the seven-day activity measurement period, the participants were asked to continue their normal daily lives while wearing an accelerometer and filling in a diary. The participants were given free tickets to visit SuperPark Jyväskylä on a minimum of one and a maximum of four days during the measurement week. After the measurement period, the participants were given an additional three free tickets to SuperPark, SuperPark-branded socks, and a SuperPark sticker as a gift for participation.

### 2.2. The SuperPark Concept

SuperPark Ltd. is a limited company with 13 parks in Finland and one in Hong Kong, China. The SuperParks are located in large indoor halls and include areas for different physical activity concepts. Examples of these concepts include Adventure Area (including Obstacle Walls, Zip Wires, Slides, Playtowers, Pedal Car Racing Track, etc.), Game Arena (including Ice Hockey Shooting, Baseball Hitting, Superpinball, etc., with radar and other technology to measure performance), and Freestyle Hall (including Gymnastic Area, Skate and Scoot World, Trampoline Platform, etc.). All activities can be seen at https://superpark.fi/en/activities/ (referred 19.5.2018). There are several SuperPark coaches present in the parks to help, teach, and encourage children to perform the different physical activities. SuperPark sells one-day tickets or monthly and yearly passes.

### 2.3. Laboratory Measurements

The participants arrived at the University of Jyväskylä research laboratory in a fasted state. After measuring participants’ height, their weight, lean and fat mass were measured with an InBody device (InBody 720, Biospace Ltd., Seoul, Korea). After the measurements, the participants were given a breakfast package. A Fibion accelerometer (worn in a thigh strap) was either attached by a researcher right away or was given to the participant to be worn at home when starting the seven-day period. Instructions for the measurements and an activity diary were given.

### 2.4. Accelerometer Measurements

Daily sitting and physical activity were measured with a thigh-worn accelerometry-based device (20 g, L = 30 mm, W = 32 mm, T = 10 mm; Fibion Inc, Jyväskylä, Finland). The Fibion device was worn in an elastic thigh strap, which was fit snuggly on the participants’ right thigh anteriorly with a Velcro attachment. The Fibion device measures raw acceleration on three axes (internal sampling rate 12.5 Hz). Because of the wear position on the thigh, the device orientation and impact data are used to estimate postures and activity classes (see below for further details). The Fibion device has no buttons or display and can operate for around 30 days on full charge condition.

The participants kept a diary for time when going to bed, time when waking up, school or work time, any physical activity start and end time, including the SuperPark time, bathing times or other times when the monitors were detached, and any abnormal days or behaviors. Participants were instructed to detach the monitors for nights, bathing, and any water-based activities and to put them back on immediately after these occasions.

Data from the Fibion Devices were uploaded from the devices to the manufacturer’s web-browser-based online service (www.fibion.com/upload) and the participants’ weight, height, age, and sex were submitted to the service. The service analyses the data and provides the analysis report including day-by-day results for non-wear, sitting, long (>30 min) continuous sitting periods, standing, light and moderate walking, cycling, vigorous activity, as well as light (sitting <3 METs), moderate (3–6 METs), and vigorous (>6 METs) activity times. The results for sitting, long sitting periods, light (including standing), and MVPA are reported. The Fibion device gives similar results for sitting, long sitting periods, light activity time, and MVPA as compared to video recordings (unpublished results submitted for review). For research purposes, Fibion provides access to the Fibion RT-tool, which provides hour-by-hour results in a spreadsheet format, which was used for further analysis in Microsoft Excel.

A minimum of one day with and one day without a SuperPark visit was required for a participant to be included in the analyses. Non-wear time was defined as >30 min periods when the device remained still. The days with a minimum of eight hours sitting and activity data were included in the analyses, and the final results are presented as an average of complete days. The diaries were used to analyze the SuperPark periods, and average of corresponding periods at the same time of day on the days without a SuperPark visit. For example, if a SuperPark visit from 3.00 p.m. to 5.30 p.m. was reported, an average result of the corresponding time periods from the other days were analyzed as the reference periods. In case of several SuperPark visits, the results were averaged. Complete accelerometer data for the whole week was obtained from 12 participants, whereas data was missing from 3 participants (device battery ran out before the first SuperPark visit *n* = 2, did not return diary *n* = 1). Additionally, results for weekdays (complete *n* = 6, both SuperPark and reference days not complete *n* = 8, did not return diary *n* = 1) and weekend days (complete *n* = 7, both SuperPark and reference days not complete *n* = 7, did not return diary *n* = 1) were analyzed for those participants who had a minimum of one complete SuperPark and reference day and a period from either weekdays or weekends.

### 2.5. Questionnaires

At baseline, parents reported their children’s sitting, walking, and moderate and vigorous physical activity durations with the short form of the international physical activity questionnaire (IPAQ-SF) [20]. Number of times per week and duration per each session of physical activities were asked. The resulting volume of physical activity was divided by seven days to yield an average duration of physical activity per day in a week.

### 2.6. Statistical Analyses

Statistical analyses were performed with SPSS Statistics version 24.0 (SPSS inc., Chicago, IL, USA). Data are presented as mean ± standard deviation. Background characteristics were compared between girls and boys with independent sample t-test and between those having complete vs. incomplete measurements with the Mann-Whitney U test. The Shaphiro-Wilk test revealed that the whole day and the SuperPark period accelerometer data from the whole week were normally distributed, and were analyzed with a paired sample *t*-test, with some exceptions that were analyzed with the Wilcoxon test (whole day: Long sitting period duration; SuperPark period: Non-wear time, MVPA, long sitting periods). The weekday and weekend day data were analyzed with the Wilcoxon test. Statistically significant differences were considered at *p* < 0.05.

## 3. Results

The participants were on average 10-year-old normal weight (body mass index (BMI) < 25 kg/m^2^) girls and boys (Table 1). Self-reportedly, they accumulated more than two hours walking and MVPA per day but were sitting almost six hours per day. Girls and boys had similar background characteristics. There were no background differences between those children who had complete vs. incomplete whole week, weekday, or weekend day measurements.

The recording time was on average 12.8 ± 1.5 h on 6.3 ± 1.1 days without differences between the reference and the SuperPark days (Table 2). On average, the children spent 3.3 ± 1.2 h per visit in SuperPark on 0.5 ± 0.5 weekdays and 1.1 ± 0.5 weekend days. Four girls and one boy visited SuperPark on both weekdays and weekend days, whereas two girls and four boys visited SuperPark only on weekend days and one girl only on weekdays. As compared to the reference periods, when in SuperPark, the children had 0.9 h less sitting (*p* < 0.001), 0.9 h more MVPA (*p* = 0.002) and 0.5 h less long sitting periods (*p* = 0.002). During the whole day when having visited SuperPark the children had 1.0 h less sitting (*p* = 0.008) and 0.8 h more MVPA (*p* = 0.017).

Data for 4.8 ± 0.4 weekdays with a minimum of one SuperPark and a reference day was obtained from 5 girls and 1 boy. The children spent on average 2.7 ± 0.5 h in SuperPark on 1.0 ± 0.0 weekdays. As compared to the reference periods, the children had 0.9 h less sitting (*p* = 0.028), 0.2 h less light physical activity (*p* = 0.046), 1.2 h more MVPA (*p* = 0.028) and 0.5 h less long sitting periods (*p* = 0.028) when in SuperPark. During the whole weekdays the children had 0.2 h less light physical activity (*p* = 0.028) and 1.2 h more MVPA (*p* = 0.028) on the days when they visited SuperPark as compared to the reference days.

Data for 2.0 ± 0.0 weekend days with at least one SuperPark and a reference day was obtained from 5 girls and 2 boys. The children spent on average 2.9 ± 0.7 h per their one visit in SuperPark on one weekend day. As compared to the reference periods, during their time in SuperPark, the children had 0.5 h less sitting (*p* = 0.018) and 0.8 h more MVPA (*p* = 0.028). There were no differences between sitting or physical activity during the whole weekend days.

## 4. Discussion

Commercial indoor activity parks provide an entertaining physical activity option for children, but their potential to improve physical health depends on whether visiting such a park can reduce sitting and increase physical activity during the whole day. This study looked at whether children visiting a commercial indoor activity park, SuperPark, reduce their sitting time and increase their physical activity time during the visits and the whole day as compared to their usual days. Children spent on average over three hours in SuperPark on their one visit, of which on average 1.4 h was MVPA. Therefore, on one visit, the children exceeded the landmark of daily 60 min of MVPA, which the World Health Organization recommends for all children and youth to get health benefits [21]. On the days when visiting SuperPark, the children had on average one hour less sitting and 0.8 h more MVPA as compared to their usual days. These results suggest that a commercial indoor activity park can engage children for long physical activity sessions, and the increased total physical activity time is not compensated for by being more sedentary outside of the visit. If children continue to visit SuperPark, they will get health benefits.

The commercial nature of SuperPark needs to be considered when evaluating the effects on children’s physical activity. An important factor to consider is the cost of a visit. In this study, SuperPark provided free tickets to the participants, but usually visitors need to pay for a visit. Therefore, the real-life scenario and effects may differ from those observed in this study. It is important to note that participating in organized sports usually has costs, and the costs are a barrier for participation [22]. However, the commercial nature may also have several benefits. For example, it may be that once having paid for a visit, one wants to get value for the money by staying in the park for a long time, which may further increase the total activity time. The income also enables the developing of entertaining physical activity concepts and hiring of the coaches, which may be reasons for the observed high physical activity time during the visits. In respect to organized sports, autonomy-supportive coaching is associated with higher MVPA during the whole day in children [23]. Although we did not assess if the children were in contact with the coaches in SuperPark, this illustrates the potential of coaching on children’s motivation and physical activity both during and outside of training. The costs may also affect the long-term adherence. On one hand the costs may prevent some visitors from continuing visiting the park in the future. On the other hand, having paid for a long-term subscription (like a season fee in football or a monthly pass to SuperPark) may increase the long-term adherence and the total activity time. It should be noted that several free forms of physical activity might have a similar potential in increasing total physical activity. Examples include playing in a park or participating in playing with dads who at the same time serve as coaches. Both the positive and the negative effects of commercial physical activity supply on the total activity in children should be studied further.

A typical supervised exercise training session differs from a SuperPark visit in several ways. A supervised training typically has a fixed duration (e.g., one hour) and the exercises are often pre-determined. The accumulated total volume of exercise depends on the intensity and duration of one training session. Although many popular team sports have been considered to be of vigorous intensity, only about a half or less time during one session is performed at moderate-to-vigorous intensity, whereas the rest of the time is sedentary or light activity [24,25,26,27]. In SuperPark, the children can choose the exercises and the duration freely. In a previous study, children were found to be more active during free than structured activities [28]. In the present study, the children spent about half of their visit in MVPA, which is a similar proportion as recorded in team sports [25,27]. However, the average visit duration was more than three hours and resulted in a three-times longer total duration of MVPA per session as compared to the shorter organized training sessions measured in previous studies. Consequently, visiting SuperPark increased total daily MVPA by about 48 min as compared to reference days, which is slightly more than a 30-min increase observed in a previous study [25]. Therefore, the duration of one training session appears to be a determining factor in the effect on total daily physical activity time. Based on the present findings, it appears that children are willing to spend several hours being physically active in SuperPark.

Daily MVPA is not necessarily associated with less sedentary time in children. Daily sedentary time is similar in children who do and do not take part in organized sports [29]. Reasons for this asymmetry include that MVPA takes place at different places, at different times of the day, and is determined by different socioeconomic factors than sedentary time, therefore, it is not a direct replacement for sedentary time [13,16,30,31]. However, in the present study, the children replaced one hour of sitting with one hour of MVPA following visits to SuperPark, suggesting that these behaviors can directly replace each other within an individual’s daily life. These findings are supported by other studies using a within-individual design, where increases in MVPA have been associated with decreased sedentary time during the same or the following days [18,32]. These findings are in contrast to studies in adults, where sedentary time and MVPA time are independent behaviors within individual’s daily life and following participation in physical activities [33,34,35]. This is an important consideration for interventions, since a behavioral physical activity intervention could also be used to decrease sedentary time in children. On the other hand, intervention studies assessing the independent health effects of physical activity should consider the change in a mixture of other behaviors during the whole day [36,37]. For example, walking about can be light activity but important for children for gaining proficient motor coordination [38].

The strengths of this study include a within-participant design, objective measurement of the sitting posture (not merely sedentary time), and physical activity time in an ecological setup. Measuring the actual sitting posture based on thigh position has a superior validity as compared to sedentary behavior data obtained with waist-worn monitors [39]. However, the Fibion device has not been validated for children, which is a limitation. Another limitation is that because the participants were recruited from SuperPark visitors, they may have been more interested in SuperPark than a more general population. However, in the within-participant design, each participant served as his/her own control, which made it possible to compare the effects to reference days (not cross-sectionally to other individuals). Future studies should test whether these findings are generalizable to children having not visited SuperPark before and whether the observed effects endure. The small sample size prevented comparison of boys and girls. The reason for data loss from weekdays and weekend day’s analyses is obviously that the participants could select freely the days when they visited SuperPark. Many participants visited SuperPark on both weekend days and not during weekdays, which made comparisons within weekdays and weekend days impossible. However, several statistically significant findings prove that the sample was big enough for the primary purpose of this study. In addition, there may be other reasons for the significant increase in MVPA following a visit to SuperPark on weekdays but not on weekends. The weekday physical activity patterns during school hours are largely similar between children, but differences occur during leisure time after school [40]. However, during weekends, the activity patterns differ between children throughout the whole day [40]. Therefore, a change in leisure time activities can be more easily detected during weekdays because there is less noise during the whole day. Larger sample sizes should be used to test if physical activity interventions have a larger potential to increase physical activity on weekdays vs. weekends.

## 5. Conclusions

Commercial physical activity supply is likely to increase. Commercial indoor activity parks can provide children with entertaining physical activity content, but the health effects of visiting such a park depends on if the total daily physical activity is increased without compensation in sitting time. In this study, children spent more than three hours in SuperPark on their one visit, of which almost a half was MVPA. On the days when they visited SuperPark, children had one hour less sitting and 0.8 h more MVPA as compared to their usual days without a visit. These results suggest that parents taking their children to SuperPark can expect decreases in the children’s sitting time and increases in MVPA during the whole day, which may lead to long-term health benefits. The benefits (like the entertaining content and support from coaches) and disadvantages (like if the costs prevent long-term adherence) of visiting SuperPark should be studied further to assess the generalizability of these findings.

## Figures and Tables

**Table 1:** Participant characteristics.

	Boys	Girls	Total	*p*-Value
*n*	7	8	15	
Age (years)	11.0 ± 1.8	9.7 ± 2.0	10.3 ± 1.9	0.212
Height (cm)	148.2 ± 8.0	141.3 ± 14.1	144.5 ± 11.8	0.279
Weight (kg)	42.5 ± 5.7	39.7 ± 14.0	41.0 ± 10.7	0.638
BMI (kg/m^2^)	19.4 ± 2.4	19.3 ± 3.6	19.3 ± 3.0	0.966
Body fat (%)	21.1 ± 9.3	21.0 ± 7.4	21.0 ± 8.0	0.970
Self-reported sitting time (^1^ h/d)	4.5 ± 2.3	6.4 ± 1.7	5.8 ± 2.0	0.139
Self-reported walking time (h/d)	0.5 ± 0.7	1.5 ± 1.1	1.2 ± 1.1	0.156
Self-reported moderate activity time (h/d)	1.9 ± 2.4	0.7 ± 0.4	1.1 ± 1.5	0.160
Self-reported vigorous activity time (h/d)	0.2 ± 0.2	0.3 ± 0.4	0.3 ± 0.3	0.328

^1^ h/d, hours per day. BMI = body mass index.

**Table 2:** Objectively measured sitting and physical activity time for the reference and SuperPark days and the reference and SuperPark periods during the whole week, weekdays, and weekends.

	Whole Day		SuperPark Period		*p*-Values	
	Reference	SuperPark	Reference	SuperPark	Whole Day	SuperPark Period
Whole week (Girls *n* = 7 and boys *N* = 5)						
Days (N)	4.8 ± 1.4	1.6 ± 0.5	4.8 ± 1.4	1.6 ± 0.5		
Weekdays (N)	4.0 ± 1.0	0.5 ± 0.5	4.0 ± 1.0	0.5 ± 0.5		
Weekend days (N)	0.8 ± 0.6	1.1 ± 0.5	0.8 ± 0.6	1.1 ± 0.5		
Recording time (h)	12.9 ± 1.2	12.7 ± 1.9	3.3 ± 1.2	3.3 ± 1.2	0.693	NA
Non-wear time (h)	10.9 ± 1.5	11.3 ± 1.9	**0.2 ± 0.4**	**0.0 ± 0.0**	0.427	**0.015**
Sitting (h)	**6.8 ± 1.1**	**5.8 ± 0.9**	**1.6 ± 0.3**	**0.7 ± 0.3**	**0.008**	**0.000**
Light (h)	3.7 ± 0.9	3.4 ± 1.2	0.9 ± 0.3	1.1 ± 0.7	0.393	0.162
^1^ MVPA (h)	**2.2 ± 0.8**	**3.0 ± 1.0**	**0.5 ± 0.4**	**1.4 ± 0.6**	**0.017**	**0.002**
^2^ Long sitting (h)	1.8 ± 0.6	1.2 ± 1.1	**0.5 ± 0.2**	**0.0 ± 0.1**	0.124	**0.002**
Weekdays (Girls *n* = 5 and boys *n* = 1)						
Days (N)	3.8 ± 0.4	1.0 ± 0.0	3.8 ± 0.4	1.0 ± 0.0		
Recording time (h)	13.5 ± 1.1	12.9 ± 2.1	2.7 ± 0.5	2.7 ± 0.5	0.345	NA
Non-wear time (h)	10.1 ± 1.6	11.1 ± 2.1	0.0 ± 0.1	0.0 ± 0.0	0.345	0.345
Sitting (h)	7.3 ± 0.9	5.9 ± 1.0	**1.6 ± 0.4**	**0.7 ± 0.2**	0.173	**0.028**
Light (h)	**3.9 ± 0.8**	**3.1 ± 1.1**	**0.7 ± 0.2**	**0.5 ± 0.2**	**0.028**	**0.046**
^1^ MVPA (h)	**2.2 ± 0.9**	**3.3 ± 0.7**	**0.3 ± 0.1**	**1.5 ± 0.6**	**0.028**	**0.028**
^2^ Long sitting (h)	2.0 ± 0.6	1.3 ± 1.0	0.6 ± 0.3	**0.1 ± 0.2**	0.116	**0.028**
Weekend days (Girls *n* = 5 and Boys *n* = 2)						
Days (N)	1.0 ± 0.0	1.0 ± 0.0	1.0 ± 0.0	1.0 ± 0.0		
Recording time (h)	13.3 ± 0.9	12.4 ± 1.6	2.9 ± 0.7	2.9 ± 0.7	1.000	NA
Non-wear time (h)	10.7 ± 0.9	11.6 ± 1.6	0.0 ± 0.0	0.0 ± 0.0	1.000	0.176
Sitting (h)	7.2 ± 1.1	6.2 ± 0.7	**1.3 ± 0.6**	**0.8 ± 0.3**	0.398	**0.018**
Light (h)	3.8 ± 1.1	3.2 ± 1.4	1.2 ± 0.6	0.9 ± 0.6	0.735	0.237
^1^ MVPA (h)	2.0 ± 0.6	2.7 ± 1.2	**0.4 ± 0.3**	**1.2 ± 0.4**	0.128	**0.028**
^2^ Long sitting (h)	2.0 ± 0.8	1.3 ± 1.4	0.4 ± 0.6	0.0 ± 0.1	0.345	0.109

^1^ MVPA, moderate-to-vigorous physical activity; ^2^ Long sitting, >30 min continuous sitting periods.
